# “Rosary of Testes”: Splenogonadal Fusion in Association with Bilateral Abdominal Testes Presenting as Polyorchidism

**DOI:** 10.1155/2015/317189

**Published:** 2015-11-29

**Authors:** Pejman Shadpour, Behkam Rezaimehr

**Affiliations:** ^1^Hasheminejad Kidney Center (HKC), Hospital Management Research Center (HMRC), Iran University of Medical Sciences (IUMS), Vanak Square, Tehran 19697, Iran; ^2^Mazandaran University of Medical Science (MazUMS), Sari 23224, Iran

## Abstract

Polyorchidism is a rare anomaly where early segmentation in the gonadal ridge can lead to the development of three or less commonly four testes in one individual. Just over 150 reports of this phenomenon exist in English medical literature. However, once confronted by the clinical finding of supernumerary gonads, one must remain mindful of other likely diagnoses involving nontesticular origin. We report on a male patient with bilaterally impalpable testes in whom splenogonadal fusion mimicked polyorchidism. By keeping such differential diagnoses in mind, surgeons are more liable to take the appropriate intraoperative course of action.

## 1. Introduction

Splenogonadal fusion is a rare congenital anomaly characterized by the presence of splenic tissue adjacent to a gonad, which usually presents as an incidental finding during exploration for inguinal hernia, cryptorchidism, or less commonly a mass [1], sometimes coinciding with infertility [2]. It has been classified into continuous and discontinuous types based on attachment to the spleen. We report on a young adult with bilateral impalpable testes in whom multiple encapsulated ovoid structures resembling multiple testes were found during laparoscopic exploration. These distinctively separate splenic bodies led up to the spleen in* significant contradiction* to the firmly held notion that only* discontinuous* tissue shall condense into free standing structures.

## 2. Case Report

A 17-year-old male was referred to our center for overdue evaluation of bilateral cryptorchidism. Past medical history was unremarkable. On general physical examination he appeared as a normally developed adult male; the scrotum was hypoplastic and empty, but stretched penile length was 13 cm, within normal limits. No gonad was palpable in the inguinal or paragenital regions. Ultrasonography did not detect any gonadal structure in the scrotum, inguinal canals, pelvis, or abdomen. No further imaging was done. Laboratory data revealed azoospermia, normal LH, normal testosterone, and minimally elevated FSH.

We scheduled the patient for laparoscopic exploration, suspecting the existence of some testicular source of androgen but with informed consent for gonadectomy on either side if necessary.

On laparoscopic evaluation we found a 35 mm long abdominal testis with normal vas, epididymis, and vessels covered by bowel at high iliac position on the right. This gonad was successfully pexed into the right hemiscrotum by combining one-stage Fowler-Stephens and Prentiss maneuvers. Taking a biopsy proved arterial flow intraoperatively and later confirmed sertoli cells only with germ cell aplasia and no evidence of intratubular germ cell neoplasia.

On the left side, however, a smaller 20 mm long gonad resembling the testis was encountered distally in the pelvis but with anomalous total disjunction from the hypoplastic vasoepididymal structures. Because there was no continuity between the left gonad and epididymis, the only blood supply to the organ was via internal spermatic vessels, making this short spermatic vascular pedicle indispensable, and Fowler orchiopexy is not an option, even if staged.

**Figure 1:**
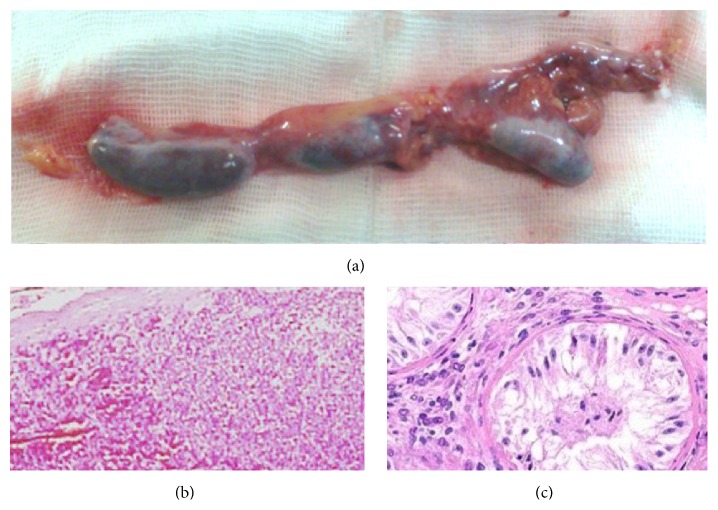
(a) The rosary of “polyorchia,” right end includes testis and epididymis; (b) splenic micrograph; (c) testis micrograph.

We began to mobilize the left testis with its meager distal attachments still connected. Moving proximally along the well-developed spermatic vascular bundle a second off-white ovoid structure resembling a third “testis” came into view. Further dissection led to yet another identical element. The rosary of three tandem structures, leading from the anomalous vasoepididymal stump, was followed cephalad and laterally by tilting the table to the right to disclose that the retroperitonealized fibrous band connecting them led into the inferolateral aspect of the lienophrenic ligament. The rosary was freed and excised (Figure 1(a)). Histopathologic evaluation revealed normal splenic tissue (Figure 1(b)) in the two cephalad ovoid structures and sertoli cell only in the smaller left testis exactly copying the finding on the right side (Figure 1(c)).

## 3. Discussion

Like polyorchidism, splenogonadal fusion is also a rare congenital anomaly. Since its first description by Bostroem in 1883 and later by Pommer in 1889 [3], about 170 cases have been reported in the English literature on PubMed. The condition occurs mostly on the left side and is arguably 16-fold more common in males [3, 4]. The phenomenon has been classified into continuous and discontinuous types based on attachment to the spleen [3]. Colonization of the phrenic ligaments by splenic cells in the first two months of gestation is believed to create the splenic processus in continuous cases [4]. Therefore, splenic tissue has characteristically been encountered as the tip of a contiguous elongation of the parent organ extending downward in the* continuous* type [5, 6]. The discontinuous type, without any attachment to the spleen proper, is thought to be just another manifestation of much more prevalent accessory spleens [3, 4].

Finding separate encapsulated units connected to the testis and epididymis distally and to the spleen cephalad—by a fibrous band alone–as exemplified by our case, is in contrast to existing literature and therefore is most interesting to look out for in the future. Perhaps this may justify further subdivision of the continuous type of splenogonadal fusion anomaly into* parenchymal* (including islands of splenic tissue) and* fibrotic* (entirely composed of connective tissue) variants in the future.

Polyorchidism and splenogonadal fusion are both very rare events; hence there is no consensus on their management.  Table 1 shows their differential characteristics. Although most case reports have ended in orchiectomy to date, but salvaging the gonad is occasionally possible in both scenarios [1, 7, 8]. In our 17-year-old case, salvaging the testis was not at all an option as explained above. We believe that awareness about both conditions and their management options can be of help during inevitable laparoscopic exploration for the nonpalpable testis, for as long as less invasive diagnostic imaging tools fall short in sensitivity and specificity [9].

## Figures and Tables

**Table 1:** Differential characteristics of splenogonadal fusion and polyorchidism.

	Splenogonadal fusion	Polyorchidism
Cases reported to date (approx.)	170	150

Laterality	Almost always left	L > R

Associated anomalies	Hernia, cryptorchidism, limb, and facial defects	Cryptorchidism

Sonographic findings	Homogeneous solid isoechoic	Homogeneous solid isoechoic

Tc sulfur colloid scan	Strong uptake	No uptake

MRI	Signal distinct from normal testis	Same as normal testis

Histology	Splenic pulp	Testicular tissue

Complications	Enlargement along with other causes of splenomegaly	Enlargement and pain with torsion or tumoral degeneration

## Abbreviations

FSH: Follicle stimulating hormone
LH: Luteinizing hormone.
